# Dynamic Traffic Assignment Model Based on GPS Data and Point of Interest (POI) in Shanghai

**DOI:** 10.3390/s21217341

**Published:** 2021-11-04

**Authors:** Xueying Song, Zheng Yang, Tao Wang, Chaoyang Li, Yi Zhang, Ganyu Chen

**Affiliations:** 1 School of Naval Architecture Ocean and Civil Engineering, Shanghai Jiao Tong University, Shanghai 200240, China; 5141419016@sjtu.edu.cn (X.S.); daredevildd@sjtu.edu.cn (Z.Y.); cyljjf@sjtu.edu.cn (C.L.); darrenzhy@sjtu.edu.cn (Y.Z.); 2 School of Mechanical Engineering, Shanghai Jiao Tong University, Shanghai 200240, China; cgy2015@alumni.sjtu.edu.cn

**Keywords:** point of interest, dynamic traffic assignment, GPS, peak hours, traffic congestion

## Abstract

Dynamic traffic flow, which can facilitate the efficient operation of traffic road networks, is an important prerequisite for the application of reasonable assignment of traffic demands in an urban road network. In order to improve the accuracy of dynamic traffic flow assignment, this paper proposes a dynamic traffic flow assignment model based on GPS trajectory data and the influence of POI. First, this paper explores the impact patterns of POI on regional road network congestion during peak hours through qualitative and quantitative analysis. Then, based on the user equilibrium theory, a dynamic traffic flow assignment model, in which the effect of POI on links is reflected using the link-node impedance function, is proposed. Finally, the accuracy of the model is validated by the GPS trajectory data and origin–destination (OD) traffic data of motor vehicles in Xuhui District, Shanghai, China. The results show that the model can be used to coordinate and optimize the traffic assignment of the regional road network under the influence of POI during peak hours and alleviate the congestion of the road network. The findings can provide a powerful reference for developing scientific and rational traffic assignment decisions and management strategies for urban road network traffic.

## 1. Introduction

With the rapid development of urban construction, traffic congestion has become a thorny issue. In 2019, 4.438 million motor vehicles were registered in Shanghai, China, with a year-on-year rise of 5.3%. However, the total length of the city’s road network increased by 0.6% year-on-year [1]. As reported, among mega cities, Shanghai has the lowest average speed of 24.29 km/h during peak hours [2]. It is urgent for Shanghai to solve the traffic congestion problem. Therefore, dynamic traffic flow assignment, which can automatically allocate spatial resources according to the travel demand based on real-time traffic information in a timely manner, has attracted attention of scholars [3,4,5,6]. By providing the optimal travel route for travellers, and effectively alleviating traffic congestion caused by blind or empirical driving, dynamic traffic flow assignment has been expected to play a critical role in ensuring smooth traffic and improving road network usage efficiency.

Dynamic traffic assignment (DTA) has received increasing attention from scholars in the field of intelligent transportation systems [7,8,9,10]. The purpose of traffic network analysis is to describe the relationship between time-varying networks and users’ travel behaviour patterns in a rational way [11], and the model analysis results can optimize network traffic assignment, alleviate urban congestion, and provide a reference for policy measures of regional road network planning in most cities in China. Merchant and Nemhauser [12] were the first to develop a study on dynamic traffic assignment using mathematical planning models, describing the system optimization model with nonconvex nonlinear programming and discretizing time. This model can be transformed into a static system optimal model for analysis based on static assumptions. Friesz et al. [13] discussed system optimal (SO) and user equilibrium (UE) for the single objective case under the assumption that the adjustment of the network from one system state to another may occur simultaneously with network changes, and this theoretical approach to optimal control is widely adopted in subsequent studies. Meng et al. [14] developed a multiclass, multimodal dynamic traffic equilibrium model that derives optimal travel routes and optimal departure times, which is beneficial for traffic control and dynamic route guidance. Zhao et al. [15] analysed the dynamic traffic assignment problem under three types of networks based on day-based incentive routing strategies using a graphical solution and extended it for general parallel networks to demonstrate the effectiveness of the scheme. In summary, the initial research on dynamic traffic assignment mainly lies in the theory, and the model building methods and iterative updates of algorithms are the focus of scholars’ attention.

With the development of dynamic traffic flow assignment research, based on increasingly sophisticated theoretical models and algorithms, scholars have gradually extended the study of dynamic traffic flow theory to traffic management and the application of models in multiple traffic modes, including private car traffic, public transportation, and slow-moving traffic [16,17,18,19,20]. Kamel et al. [21] developed a simulation-based dynamic assignment model for cars and transit that preserves the interactions between the assignment processes of two traffic modes in a large network in the Greater Toronto Area during the morning peak. The model was suitable for testing different demand management strategies that impose dynamic changes on multiple modes simultaneously. Hu et al. [22] developed a dynamic traffic assignment simulation algorithm under mixed traffic flows, considering and modelling four different physical vehicle types, including cars, buses, motorcycles, and trucks. Numerical experiments were conducted in a test network and a real city network, and the results showed that this DTA program can accurately reflect the traffic characteristics of the city. Zhang et al. [23] studied the supply-demand imbalance of an airport network system and developed an open multi-airport-oriented network flow system. This system can be used to coordinate and optimize the passenger flow and airport capacity matching problem in a multi-airport situation to fully utilize airport resources and minimize system delays. Zhang et al. [24] found that a dynamic traffic assignment system is able to estimate and predict traffic flow and speed with high accuracy during traffic congestion, and developed a method for tolling during road congestion based on this system. Experimental results in several experimental scenarios showed that the method was able to increase toll revenue compared to when online traffic data calibration was not available.

Most of the above attempts have been made focusing on the iterative update of the assignment method itself, and the validity test of the model is mostly conducted in the virtual single-vehicle road network set manually. Little literature meticulously analysed the impact of realistic factors such as POI (Point of Interest) and intersection impedance on traffic flow in the road network. In recent years, more and more studies notice static traffic impact factors such as schools, shopping malls, and parks (i.e., POIs), which can be summarized into three categories: identification of urban functional areas [25,26,27,28], analysis of the spatial distribution of a specific type of public facilities [29,30,31], and analysis of urban traffic characteristics based on POI data [22,25,32,33]. It is concluded that the impact of static traffic factors on cities is not negligible, and they have significant value for research on sustainable urban development.

In order to scientifically plan the urban traffic operation and promote the healthy development of an urban road network, this paper proposes a traffic flow assignment model based on GPS trajectory data and the influence of POI in the road network and dynamic user equilibrium theory. Firstly, based on the motor vehicle GPS trajectory data, this paper analyses the way POI affects the traffic flow of its link during peak hours and verify that it has an impact on regional road network congestion. Secondly, in order to reflect the influence of actual factors on the traffic flow of the link in the theoretical model, dynamic user equilibrium theory and link-node impedance function are introduced to propose a dynamic traffic flow assignment model. Finally, the proposed algorithm and model are verified by simulation using the motor vehicle GPS trajectory data and OD traffic data of a regional road network in Xuhui District, Shanghai. The simulation results show that, by focusing the research on the actual road conditions, the method of this paper makes the link traffic assignment results more accurate and in line with the actual traffic situation. It has implications for the evacuation and inducement of traffic flow during short peak hours, the guidance of urban regional road network traffic, and the solution of congestion problems.

## 2. Materials and Methods

Based on the congestion characteristics of the road network and travelers’ path selection behavior during peak hours, this paper utilizes a traffic flow assignment model of user equilibrium, according to which travelers tend to the path with the shortest travel time. Consider a directed graph G=(N,L), where N and A are the sets of nodes and links, respectively. For each link, flow-dependent travel costs are defined, $c_{l}(x_{l})$, ∀l∈L. A set of OD pairs, RS, and sets of alternative paths, $K^{rs}$, ∀rs∈RS, for each OD pair are also given. Model assumptions: (1) The influence of non-motorized vehicles and pedestrians is not considered. (2) The travel time perceived by the traveler is consistent with the actual situation. (3) Travelers make path choices independently without interfering with each other.

The objective function of the dynamic user equilibrium model is to minimize the sum of the costs of all links, expressed as follows:

(1) $$minZ\left(x\right)=\underset{l}{\sum }\overset{x_{l}}{\underset{0}{\int }}c_{l}\left(w\right)dw$$

where Z(x) denotes the sum of impedances of all links in the road network, i.e., the sum of travel times. $x_{l}$ is the assigned traffic flow of link l.

The first constraint can be formulated as follows:

(2) $$x_{l}=\underset{rs}{\sum }\underset{a}{\sum }f_{rs}^{a}\delta_{rs}^{al}\;\forall a\in A$$

where $f_{rs}^{a}$ denotes the flow between the origin and destination (r, s) through the path a, $\delta_{rs}^{al}$ is a 0–1 parameter; when the path a contains the link l, the value of $\delta_{rs}^{al}$ is 1, and vice versa, it is 0.

According to the flow conservation principle, the second constraint can be formulated as follows:

(3) $$q_{rs}=\underset{a}{\sum }f_{rs}^{a}\;\forall r,s$$

$q_{rs}\;$ is the travel demand between the origin and destination (r, s), which is obtained through field research, and is a known input parameter.

A variable non-negative constraint formula is given in the following equation:

(4) $$x_{l}\geq 0\;\;\;\;\;\;\;\forall l\in L\;$$

(5) $$f_{rs}^{a}\geq 0\;\;\;\;\;\;\;\forall r,s,a\;$$

The impact of POI on road traffic conditions is reflected through the impedance function, which reflects the relationship between traffic delays and loads on links and intersections. The most common impedance function is the Bureau of Public Road function (BPR), which can be expressed as follows:

(6) $$t_{a}\left(x_{a}\right)=t_{a}\left(0\right)\left[1+\alpha {\left(\frac{Q}{C}\right)}^{\beta }\right]\;$$

where:

$t_{a}\left(x_{a}\right)$: the actual time through the link a, i.e., the cost of impedance in a broad sense,

$t_{a}\left(0\right)$: the free flow travel time of link a,

Q: the traffic volume through the link a, in pcu/h,

C: the actual capacity of the link a, in pcu/h,

α,β: impedance impact parameters of the model, the recommended value of 0.15 and 4, respectively.

Urban traffic impedance consists of link impedance and node impedance, where node impedance refers to the loss incurred when a vehicle passes through an intersection, link impedance refers to the loss incurred when a vehicle passes through a link. The introduction of a reasonable impedance function is essential for path selection and POI-based traffic flow assignment. Therefore, the traffic impedance function can be expressed as

(7) $$T=T_{0}+T_{1}\;$$

where T is the traffic impedance; $T_{0}$ is the link impedance; $T_{1}$ is the node impedance.

PAN et al. [34] proposed an improved link impedance and node impedance model based on the BPR function and Webster model, which can be applied to the traffic impedance where the POI is located in this paper.

The link impedance is as follows:

(8) $$T_{0}=t_{0}\left\{1+\alpha {\left[1-{\left(1-\frac{K}{K_{j}}\right)}^{2}\right]}^{\beta }\right\}\;\;\;\;\;\;K\in \left[0,2K_{j}\right]$$

where:

$T_{0}$: link travel time,

$t_{0}$: link travel time when the traffic volume is 0,

K: average traffic density of the link,

$K_{j}$: theoretical blockage density,

α,β: impedance impact parameters, the recommended value of 0.15 and 4, respectively [11].

The node impedance is as follows:

(9) $$T_{1}=\{\begin{array}{c} \frac{9}{10}\left[\frac{c{\left(1-\lambda \right)}^{2}}{2\left(1-\lambda y\right)}+\frac{y^{2}}{2q\left(1-y\right)}\right]\;\;\;\;\;\;\;\;\;\;\;\;\;\;\;\;\;\;y\leq 0.8\\ \frac{c{\left(1-\lambda \right)}^{2}}{2\left(1-\lambda y\right)}+\frac{1.5\left(y-x_{0}\right)y}{\left(1-y\right)}\;\;\;\;\;\;\;\;\;\;\;\;\;\;\;\;\;\;\;\;\;\;y>0.8 \end{array}\;$$

where $y=\left[1-{\left(1-\frac{K}{K_{j}}\right)}^{2}\right]$, *y* denotes the saturation [34].

$T_{1}$: travel time of the node,

c: signal cycle of the intersection,

λ: green ratio,

$x_{0}$: saturation threshold value, taking the value of 0.8,

*q*: vehicle arrival rate of the link.

The value of $x_{0}$ is based on the classification of the level of service in China’s Urban Comprehensive Transportation System Planning Standards. When the degree of saturation is less than or equal to 0.8, it means the road is smooth or slightly congested, and when the degree of saturation is greater than 0.8, which means the road is congested, the saturation threshold, $x_{0}$ is 0.8.

The algorithm for solving the model in this paper is the Frank–Wolfe method. In 1956, Frank and Wolfe proposed an algorithm for solving the linear constraint problem, whose basic idea is to make a linear approximation to the objective function, then find the feasible descent direction by solving the linear programming, and make a one-dimensional search in the feasible domain along the direction, which is also called the approximate linearization method. The algorithm steps are as follows

1. Determine the initial value. Initial point $x_{0}\in S$, given an error ε>0, k=0;
2. Solve the approximate linear programming: $\min\nabla f{\left(x_{k}\right)}^{T}x,\;s.t\;x\in S$, to obtain the optimal solution $y_{k}$;
3. Construct feasible descent directions, let $d_{k}=y_{k}-x_{k}$, if $||\nabla f{\left(x_{k}\right)}^{T}x||\leq \varepsilon$, stop the computation and output $x_{k}$; otherwise go to the next step.
4. One-dimensional search: $\underset{0\leq \lambda \leq 1}{\min}f\left(x_{k}+\lambda d_{k}\right)$ to get step $\lambda_{k}$. Let $x_{k+1}=x_{k}+\lambda_{k}d_{k}$, k updated to k+1, go to the second step.

## 3. Experiments

### 3.1. Data Description

**Figure 1:**
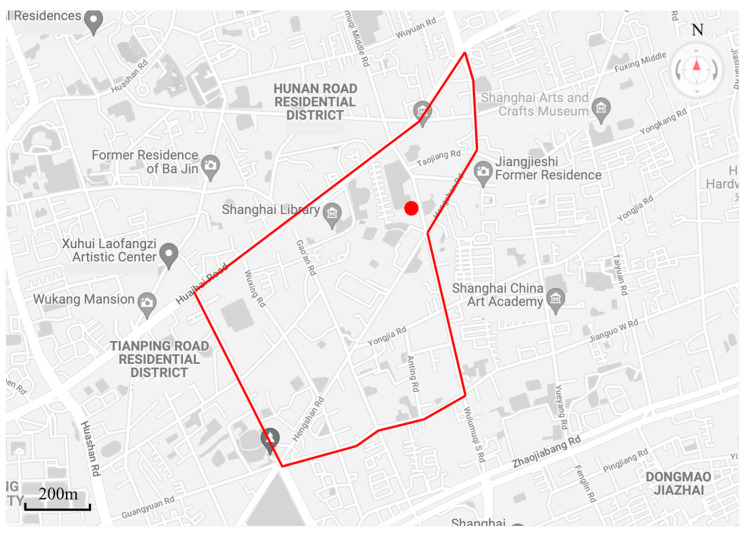
Study area and POI location.

In order to verify the validity of the application of the model in this paper to real road networks, a regional road network of about 1.3 square kilometers in Xuhui District, Shanghai, is used as the experimental road network according to the “Technical Standards of Traffic Impact Analysis of Shanghai Construction Projects”. As shown in Figure 1, the area within the red line is the experimental area of this paper. In order to simplify the analysis, the experimental road network only includes the arterial road and secondary arterial roads of the real road network, and the branch roads are not considered due to their low traffic flow; this paper only studies the travel behavior of motor vehicles, and the rest of the travel modes are not considered.

After field research in the regional road network, the research object Shanghai Wunan Kindergarten was identified as a school POI with obvious peak hour characteristics, as marked with the red dots in Figure 1. The working hours of the kindergarten are 9:00 and 16:00 on weekdays, bringing a short morning peak (8:30–9:30) and evening peak (15:30–16:30), during which parents mostly stay in front of the kindergarten for 5 to 20 min to pick up their children, causing a significant increase in road traffic. As South Wulumuqi Road, where the POI is located, is a two-way two-lane road, the short-time increase of traffic flow, vehicle stops, vehicle U-turns and other behaviors all cause significant pressure on the road, while affecting the overall traffic condition of the regional road network.

**Figure 2:**
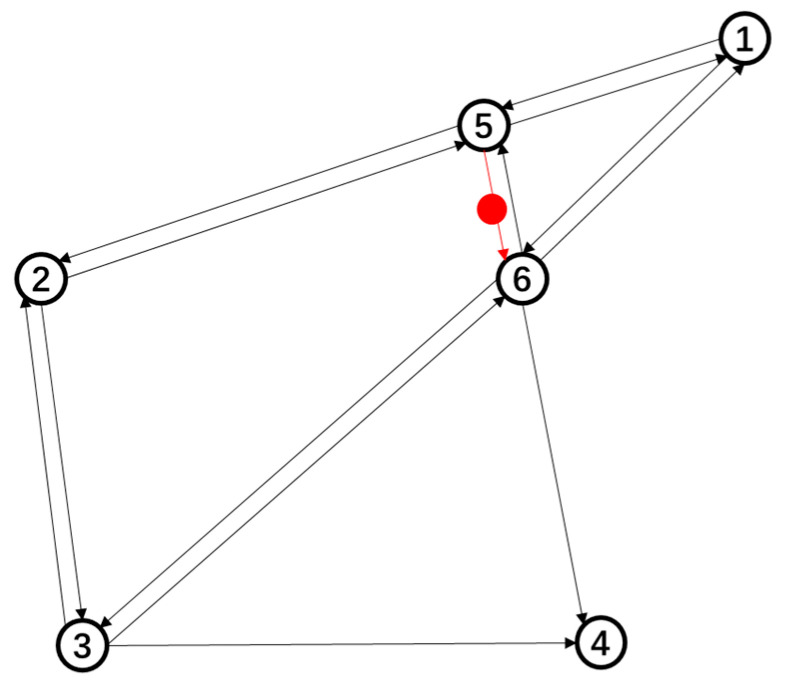
Experimental road network.

In summary, the experimental road network contains 6 nodes and 14 links, where a node represents the intersection in the actual road network and a link represents the road between two adjacent intersections. In order to simplify the road network, a schematic diagram is established as shown in Figure 2, containing a total of 19 OD pairs, and POI is located between node 5 and node 6. The red line represents the link where the POI is located, and the red dot represents the POI location.

The experimental data in this paper are obtained from the motor vehicle trajectory data provided by AMAP, and the study period is 2 November 2020, with a total of about 2.6 million rows of motor vehicle trajectory data. The data of morning peak and evening peak of the experimental road in one day are extracted, which are about 229,221 and 223,033 rows respectively.

The weekday morning and evening peaks in Shanghai are 7:30~9:30 and 16:30~18:30, and the kindergarten pick-up and drop-off times are 8:30~9:30 and 15:30~16:30. Since the kindergarten pick-up and drop-off times and the city morning peak hours overlap, and the kindergarten is located on a secondary arterial road, the traffic flow of the link has been in saturation during the city morning peak, resulting in small fluctuations in speed data and its low research value. Therefore, this paper selects the kindergarten evening peak traffic situation for analysis.

### 3.2. Performance Indexes

In order to evaluate the accuracy of the dynamic traffic flow assignment results, the performance evaluation index selected for the roadway assignment results is the travel time saving rates, where $z_{t}$ represents the actual observed value and $\hat{z_{t}}$ represents the model predicted value of the travel time.

(10) $$\mathrm{The}\;\mathrm{travel}\;\mathrm{time}\;\mathrm{saving}\;\mathrm{rate}=\frac{\left(z_{t}-\hat{z_{t}}\right)}{z_{t}}*100\%\;\;\;\;\;$$

## 4. Interpretation of Results

### 4.1. The Results of POI Impact

#### 4.1.1. Qualitative Analysis

**Figure 3:**
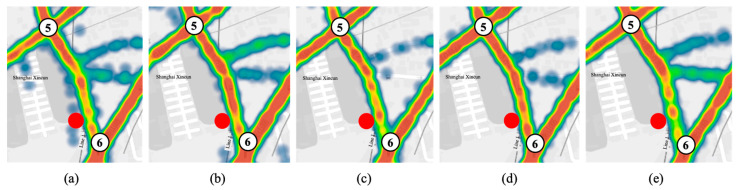
Heat map of peak hour traffic flow on the link where the POI is located: (**a**) 15:15~15:30; (**b**) 15:30~15:45; (**c**) 15:45~16:00; (**d**) 16:00~16:15; (**e**) 16:15~16:30.

The effect of the POI on the link during the evening peak is reflected by the changing pattern of the heat map. The heat map of the traffic volume in the evening peak is drawn by Power Map in Figure 3, which shows the traffic congestion of the road where the kindergarten POI (red dot) is located during the flat (15:15~15:30) and evening peak (15:30~16:30). In the figure, red indicates dense traffic, followed by yellow, and green indicates less traffic.

Table 1 shows the description of heat map of peak hour traffic flow on the link where POI is located.

The heat map from 15:15 to 15:30 is used as the control group to reflect the traffic status during the flat peak. In Figure 3, POI causes a significant increase in traffic flow on the link in a short period of time during peak hours. As a result, it has an impact on the regional road network congestion.

#### 4.1.2. Qualitative Analysis

**Figure 4:**
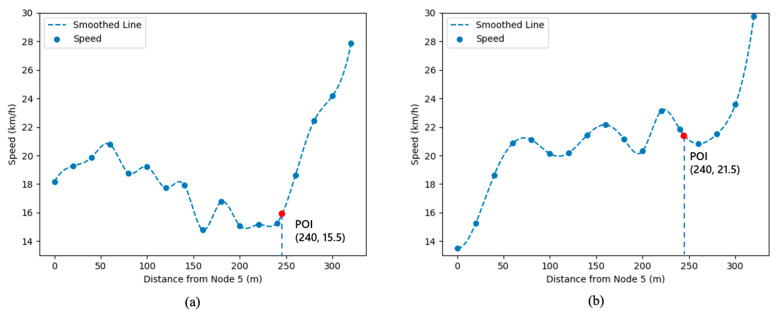
Relationship chart of POI link location and traffic speed: (**a**) 15:15~15:30; (**b**) 15:30~15:45.

By analyzing the relationship between speed and location, the effect of the POI on the speed of the link during the peak hours is explored. Figure 4 shows the speed variation graph of the link where the kindergarten POI is located during the flat and evening peak hours: the coordinates (0, 0) representing the location of the northern intersection of the link (node 5) and the coordinates (320, 0) representing the location of the southernmost intersection of the link (node 6). The total length of the horizontal axis is the length of the link where the POI is located, and the average speed of motor vehicles is calculated by taking every 20 m as a node and plotted in Figure 4. It is obvious that the impact of POI on the average speed of motor vehicles on the link is almost negligible during the flat peak hours (Figure 4a). During the evening peak hours (Figure 4b), when the motor vehicle position is north of the POI, the speed decreases the closer to the POI, indicating that the pick-up flow of parents causes congestion on the roadway. On the south side of the POI, the motor vehicle speed increases significantly and approaches the free-flow speed of the link, indicating that the road congestion is relieved after the vehicles move away from the POI. In summary, the POI causes a significant reduction in the speed of vehicles on the link during the peak hours, resulting in congestion.

### 4.2. The Results of User Equilibrium Model

In this paper, the dynamic traffic flow assignment was carried out based on the experimental road network data from 15:30 to 16:30 on 2 November 2020, and the model results are shown in Table 2, which reflects the information of 14 links. From the number of roadway traffic assignment, the traffic flow on the link from east to west is larger, but the traffic flow on the link from west to east direction is smaller, which is consistent with the reality that east to west is the direction of off-duty traffic. On the section where the POI is located (between node 5 and node 6), the assigned traffic results in a small amount traffic on node 5–node 6 and no traffic on node 6–node 5, indicating that the user equilibrium can be achieved and the total travel time is shortest when the POI link is a one-way lane during the evening peak hours.

Table 3 shows the information of 19 OD pairs and 66 paths, where those marked with bold font represent the paths with the shortest travel time among the multiple paths corresponding to each OD pair. When users have different OD demands, the path with the shortest travel time is selected to achieve dynamic user equilibrium. After calculation, the average travel time of the paths in the road network is reduced from 24.219 s to 18.488 s, which is a 23.7% reduction in travel time, proving that the optimization of the model has significant effects.

In order to evaluate the effectiveness of the model in this paper, the motor vehicle flow data of the regional road network from 15:30 to 16:30 on Monday, 9 November 2020, was selected as the test group because it is most similar to the subject of this paper in terms of the characteristics of traffic influencing factors, including weather, road network construction, and travel characteristics. The comparison between the predicted and actual values of traffic flow is shown in Table 4, where $y_{t}$ represents the actual observed value on 9 November and $\hat{y_{t}}$ represents the model predicted value on 2 November. Link [‘6’, ‘5’] and link [‘5’, ‘6’] are the links of the two directions of the link where the POI is located, respectively. On this POI link, the model predicted values are all much smaller than the actual values, indicating that during the peak hours, the traffic assigned to the link should be less than the actual traffic in order for the actual road network to better achieve user equilibrium. On some other arterial links, the predicted value of the assigned traffic flow is larger than the actual observed traffic flow, which shares the pressure of the POI link during the peak hours, and the regional road network is closer to the user equilibrium.

**Figure 5:**
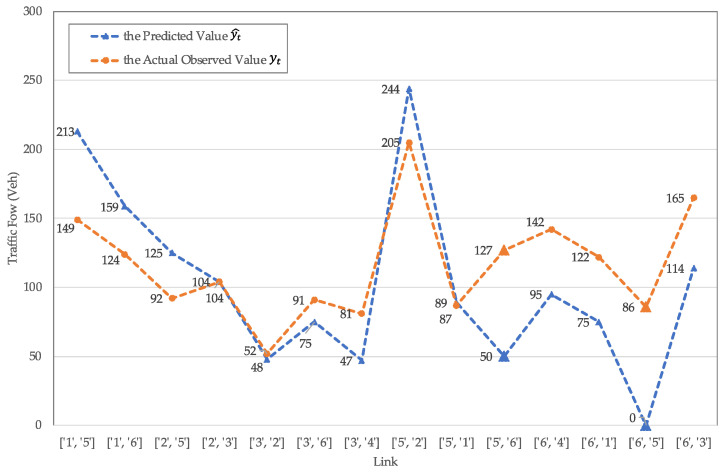
The comparison between the predicted and actual values of traffic flow.

Figure 5 shows the comparison between the predicted and actual values of traffic flow more intuitively through a line chart.

**Figure 6:**
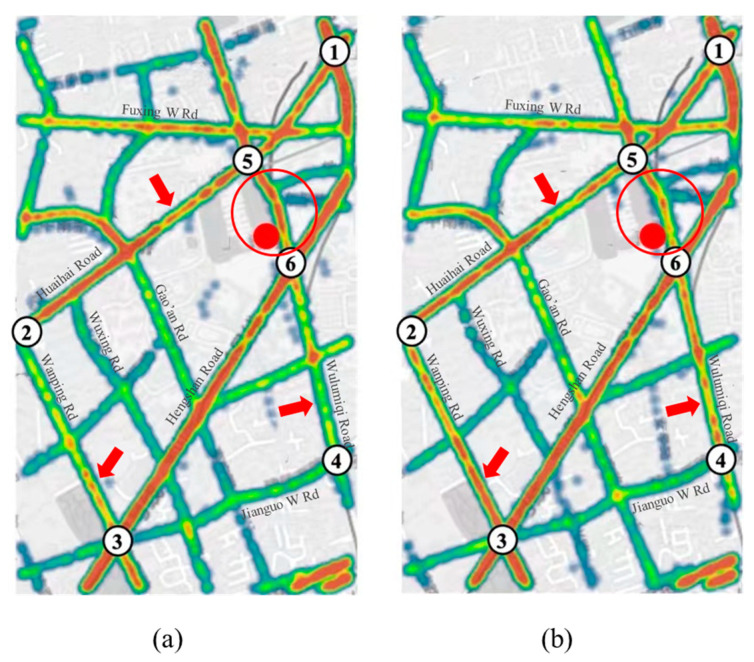
Heat map of regional road network traffic flow assignment at 15:30~15:45 on 2 November 2020: (**a**) Road network; (**b**) Forecast results.

In order to visually demonstrate the role of the model in relieving the congestion of the regional road network, the road network data from 15:30 to 15:45 on 2 November 2020, and the traffic assignment results of the model are selected for comparison and displayed in the heat map. Figure 6a shows the status quo road network, and Figure 6b shows the model results. The link of POI in the red circle in Figure 6a has more traffic flow than Figure 6b, and the link marked by the red arrow represents that Figure 6a has less traffic flow than Figure 6b. It shows that the traffic flow of the link where the POI is located should be reduced after the traffic flow assignment, while other links with high capacity should bear more traffic loads. In the link where the POI is located, the red area of the model result is significantly reduced compared with the status quo, indicating that the traffic assignment result relieves the congestion of the POI link effectively. In other links of the regional road network, some of the red area of the model result increase and the others decrease, because based on the conservation of traffic flow in the regional road network, the model assigns the status quo traffic flow of the POI link to other links in order to achieve the overall user equilibrium of the urban road network.

## 5. Conclusions

This paper proposes a dynamic traffic flow assignment model based on the influence of POI points. The model introduces dynamic user equilibrium theory and node–link impedance function, which makes the traffic flow assignment results not only fit the actual road conditions but also help to alleviate the congestion of the urban regional road network. Firstly, in order to explore the impact mode of POI on the traffic conditions of the links, this paper draws a heat map of the link traffic through qualitative analysis (Figure 3), which is helpful to visualize the phenomenon of the traffic flow around the POI increasing and dissipating during peak hours. Secondly, this paper adopts a more accurate quantitative analysis to obtain the relationship between POI link location and traffic speed, which shows that POI significantly affects the motor vehicle speed of the link where it is located. Both qualitative and quantitative analyses prove that POI is an influential factor that cannot be ignored when studying dynamic traffic flow distribution. Finally, this paper extracts model input parameter data from motor vehicle trajectory data and field research data, including road network attributes (road network structure, link connection mode), link attributes (capacity, length, free-flow travel time, OD pair, OD demand), etc. The results of the model show that the traffic flow of the link where the POI is located should be appropriately reduced during peak hours and that the traffic flow should be assigned to other links with higher traffic capacity so as to share the traffic pressure of the POI link. The results show that the paper has some implications for the evacuation and inducement of traffic flow during short peak hours. The model can be used to improve the accuracy of POI-based roadway traffic flow prediction and coordinate and optimize the traffic assignment of the regional road network during the peak hours of static traffic points, thus reducing the congestion of the road network and the travel time of users. The results of the study can guide traffic management departments to make targeted management decisions on the refinement of road networks and have significance for the study of high-density regional networks in large cities.

The paper has presented some initial results concerning the combination of the impact of POI and traffic assignment, but there are still some limitations. This paper analyzes the traffic flow on the secondary arterial road where the POI is located, but the impact of the POI can spread to other non-adjacent links when the POI link is saturated with traffic flow during peak hours. The impact of POI on the regional road network in saturation can be explored in subsequent studies. In addition, the impact of POI entrance/exit direction and extreme conditions on the link is a point of interest for future research. Finally, traffic flows may be affected by weather, traffic accidents, and other factors. How to use such auxiliary information to improve the model prediction precision will also be a focus in future studies.

## Figures and Tables

**Table 1:** Description table of heat map of peak hour traffic flow on the link where POI is located.

Figure 3	Flow	Congestion Level	Description
(a)	33	Light	The red area of the link is small, and the road congestion is light.
(b)	65	Heavy	The red area of the link is increasing, which means that parent pick-up vehicles are gathering.
(c)	52	Heavy	The red area of the link decreases, because the parents’ pick-up vehicles are parked in front of the kindergarten waiting for the children to be released. The speed is null and is not counted in the track data, and this type of data points is not shown on the heat map.
(d)	71	Heavy	The red area of the link increases as most of the parents have received their children. There is a short period of congestion caused by them leaving the link.
(e)	46	Light	The red area of the link decreases, and the road is reopened.

**Table 2:** Results of link information after traffic assignment.

No. of Link	Link	Length (km)	Free Time (s)	Capacity	Flow	V/C
1	[‘1’, ‘5’]	0.41	0.0068	2000	213	0.106
2	[‘1’, ‘6’]	0.62	0.0103	2000	159	0.08
3	[‘2’, ‘5’]	0.84	0.014	2000	125	0.062
4	[‘2’, ‘3’]	0.64	0.0107	1000	104	0.104
5	[‘3’, ‘2’]	0.64	0.0107	1000	48	0.048
6	[‘3’, ‘6’]	0.93	0.0155	2000	75	0.038
7	[‘3’, ‘4’]	0.67	0.0112	1000	47	0.047
8	[‘5’, ‘2’]	0.84	0.014	2000	244	0.122
9	[‘5’, ‘1’]	0.41	0.0068	2000	89	0.044
10	[‘5’, ‘6’]	0.32	0.0053	1000	50	0.05
11	[‘6’, ‘4’]	0.55	0.0092	2000	95	0.048
12	[‘6’, ‘1’]	0.62	0.0103	2000	75	0.037
13	[‘6’, ‘5’]	0.32	0.0053	1000	0	0
14	[‘6’, ‘3’]	0.93	0.0155	2000	114	0.057

**Table 3:** Results of paths and travel time information after traffic assignment.

No. OD Pair	OD Pairs	Demand	No. of Path	Time [s]	Paths
0	[‘1’, ‘1’]	0	0	0	[‘1’]
1	[‘1’, ‘2’]	103	**1**	**0.031**	**[‘1’, ‘5’, ‘2’]**
			2	0.062	[‘1’, ‘5’, ‘6’, ‘3’, ‘2’]
			3	0.0465	[‘1’, ‘6’, ‘5’, ‘2’]
			4	0.0465	[‘1’, ‘6’, ‘3’, ‘2’]
2	[‘1’, ‘3’]	114	5	0.0465	[‘1’, ‘5’, ‘2’, ‘3’]
			6	0.0465	[‘1’, ‘5’, ‘6’, ‘3’]
			7	0.062	[‘1’, ‘6’, ‘5’, ‘2’, ‘3’]
			**8**	**0.031**	**[‘1’, ‘6’, ‘3’]**
3	[‘1’, ‘4’]	45	9	0.0775	[‘1’, ‘5’, ‘2’, ‘3’, ‘6’, ‘4’]
			10	0.062	[‘1’, ‘5’, ‘2’, ‘3’, ‘4’]
			11	0.0465	[‘1’, ‘5’, ‘6’, ‘4’]
			12	0.062	[‘1’, ‘5’, ‘6’, ‘3’, ‘4’]
			**13**	**0.031**	**[‘1’, ‘6’, ‘4’]**
			14	0.0775	[‘1’, ‘6’, ‘5’, ‘2’, ‘3’, ‘4’]
			15	0.0465	[‘1’, ‘6’, ‘3’, ‘4’]
4	[‘1’, ‘5’]	110	**16**	**0.0155**	**[‘1’, ‘5’]**
			17	0.031	[‘1’, ‘6’, ‘5’]
			18	0.062	[‘1’, ‘6’, ‘3’, ‘2’, ‘5’]
5	[‘2’, ‘1’]	44	**19**	**0.031**	**[‘2’, ‘5’, ‘1’]**
			20	0.0465	[‘2’, ‘5’, ‘6’, ‘1’]
			21	0.0465	[‘2’, ‘3’, ‘6’, ‘1’]
			22	0.062	[‘2’, ‘3’, ‘6’, ‘5’, ‘1’]
6	[‘2’, ‘2’]	0	23	0	[‘2’]
7	[‘2’, ‘3’]	60	24	0.062	[‘2’, ‘5’, ‘1’, ‘6’, ‘3’]
			25	0.0465	[‘2’, ‘5’, ‘6’, ‘3’]
			**26**	**0.0155**	**[‘2’, ‘3’]**
8	[‘2’, ‘4’]	2	27	0.062	[‘2’, ‘5’, ‘1’, ‘6’, ‘4’]
			28	0.0775	[‘2’, ‘5’, ‘1’, ‘6’, ‘3’, ‘4’]
			29	0.0465	[‘2’, ‘5’, ‘6’, ‘4’]
			30	0.062	[‘2’, ‘5’, ‘6’, ‘3’, ‘4’]
			31	0.0465	[‘2’, ‘3’, ‘6’, ‘4’]
			**32**	**0.031**	**[‘2’, ‘3’, ‘4’]**
9	[‘2’, ‘5’]	51	**33**	**0.0155**	**[‘2’, ‘5’]**
			34	0.062	[‘2’, ‘3’, ‘6’, ‘1’, ‘5’]
			35	0.0465	[‘2’, ‘3’, ‘6’, ‘5’]
10	[‘3’, ‘1’]	75	36	0.0465	[‘3’, ‘2’, ‘5’, ‘1’]
			37	0.062	[‘3’, ‘2’, ‘5’, ‘6’, ‘1’]
			**38**	**0.031**	**[‘3’, ‘6’, ‘1’]**
			39	0.0465	[‘3’, ‘6’, ‘5’, ‘1’]
11	[‘3’, ‘2’]	18	**40**	**0.0155**	**[‘3’, ‘2’]**
			41	0.062	[‘3’, ‘6’, ‘1’, ‘5’, ‘2’]
			42	0.0465	[‘3’, ‘6’, ‘5’, ‘2’]
12	[‘3’, ‘3’]	0	43	0	[‘3’]
13	[‘3’, ‘4’]	45	44	0.0775	[‘3’, ‘2’, ‘5’, ‘1’, ‘6’, ‘4’]
			45	0.062	[‘3’, ‘2’, ‘5’, ‘6’, ‘4’]
			46	0.031	[‘3’, ‘6’, ‘4’]
			**47**	**0.0155**	**[‘3’, ‘4’]**
14	[‘3’, ‘5’]	30	**48**	**0.031**	**[‘3’, ‘2’, ‘5’]**
			49	0.0465	[‘3’, ‘6’, ‘1’, ‘5’]
			**50**	**0.031**	**[‘3’, ‘6’, ‘5’]**
15	[‘5’, ‘1’]	45	51	0.062	[‘5’, ‘2’, ‘3’, ‘6’, ‘1’]
			**52**	**0.0155**	**[‘5’, ‘1’]**
			53	0.031	[‘5’, ‘6’, ‘1’]
16	[‘5’, ‘2’]	99	**54**	**0.0155**	**[‘5’, ‘2’]**
			55	0.062	[‘5’, ‘1’, ‘6’, ‘3’, ‘2’]
			56	0.0465	[‘5’, ‘6’, ‘3’, ‘2’]
17	[‘5’, ‘3’]	42	**57**	**0.031**	**[‘5’, ‘2’, ‘3’]**
			58	0.0465	[‘5’, ‘1’, ‘6’, ‘3’]
			**59**	**0.031**	**[‘5’, ‘6’, ‘3’]**
18	[‘5’, ‘4’]	50	60	0.062	[‘5’, ‘2’, ‘3’, ‘6’, ‘4’]
			61	0.0465	[‘5’, ‘2’, ‘3’, ‘4’]
			62	0.0465	[‘5’, ‘1’, ‘6’, ‘4’]
			63	0.062	[‘5’, ‘1’, ‘6’, ‘3’, ‘4’]
			**64**	**0.031**	**[‘5’, ‘6’, ‘4’]**
			65	0.0465	[‘5’, ‘6’, ‘3’, ‘4’]
19	[‘5’, ‘5’]	0	66	0	[‘5’]

**Table 4:** Prediction performances of the dynamic traffic assignment model.

Link	[‘1’, ‘5’]	[‘1’, ‘6’]	[‘2’, ‘5’]	[‘2’, ‘3’]	[‘3’, ‘2’]	[‘3’, ‘6’]	[‘3’, ‘4’]	[‘5’, ‘2’]	[‘5’, ‘1’]	[‘5’, ‘6’]	[‘6’, ‘4’]	[‘6’, ‘1’]	[‘6’, ‘5’]	[‘6’, ‘3’]
$\hat{y_{t}}$	213	159	125	104	48	75	47	244	89	50	95	75	0	114
$y_{t}$	149	124	92	104	52	91	81	205	87	127	142	122	86	165

## Data Availability

This work was supported in part by Joint Laboratory for Future Transport and Urban Computing of Amap.
